# Health-Related Quality Of Life, Uncertainty, ‎and Anxiety among Patients with Chronic Obstructive Pulmonary Disease

**DOI:** 10.12688/f1000research.51936.1

**Published:** 2021-05-26

**Authors:** Nazih Abu Tabar, Mohammad Al Qadire, Imad Thultheen, Jafar Alshraideh

**Affiliations:** 1 Nursing Department, Ministry of Health, Amman, Jordan; 2 College of Nursing, Sultan Qaboos University, Muscat, Oman; 3 Faculty of Nursing, Al Al-Bayt University, Mafraq, 25113, Jordan; 4 Nursing Department, Faculty of Medicine and Health Sciences, An-Najah National University, Nablus, Palestinian Territory; 5 School of Nursing, University of Jordan, Amman, 11942, Jordan

**Keywords:** COPD, St. George ‎Respiratory Questionnaire, Jordan, Anxiety, quality of life, uncertainty

## Abstract

Patients’ with Chronic Obstructive Pulmonary Disease suffer from serious respiratory symptoms that increase anxiety, stress, and uncertainty, and affect quality of life. The aim of this study was to assess level of anxiety, uncertainty, and health related quality of life (HRQoL) among COPD patients in Jordan. Correlational cross-sectional survey design was used to collect data from 153 COPD patients. ‎The study was conducted at pulmonary clinics in three major referral hospitals in Jordan that provide care for COPD patients from different parts of the country. To assess HRQoL, St. George ‎Respiratory Questionnaire‎ was completed. Uncertainty and anxiety level was measured by Mishel's uncertainty of illness scale and state anxiety inventory respectively. The mean age of participants was 66.8 (SD= 10.3) and most participants were males (94.1%) with. The mean score of HRQoL was 57.9 (SD = 20.5). The mean score of participants’ level of anxiety was 38.1 (SD = 11.1). The mean score of uncertainty was 66.1 (SD= 11.1). There is a statistically significant positive relationship between HRQoL and anxiety (r =.433, p< .01), and uncertainty (r=.483, p<.01). Increased anxiety and uncertainty among COPD patients was associated with low HRQoL. Health care providers need to pay attention the effect of anxiety and uncertainty on COPD patients’ quality of life and institute appropriate management.

## Introduction

Chronic obstructive pulmonary disease (COPD) is one of the chronic irreversible respiratory diseases which result in comorbidities and a high number of annual deaths ^1^ leading to the increased burden of chronic diseases worldwide. ^2^ The burden associated with COPD is increasing as the percentage of smokers is increasing, with most deaths occur within the developing countries. ^3, 4^ COPD remains the most prevalent of chronic respiratory diseases globally among men and women, with an overall prevalence of 5.9% in 2017. ^2^ It is estimated that 3.6% of the population in the Middle East have been diagnosed with COPD and the figure reaches 11% among Jordanian male smokers. ^5, 6^ COPD and related symptoms are associated with increased anxiety and uncertainty that affect health-related quality of life (HRQoL). ^7^

HRQoL is an important outcome that should be evaluated among COPD patients. It is continuously changing as patients progress from one stage to another during the disease trajectory. ^8^ Several factors were found to negatively affect quality of life, including disease symptoms, such as difficulty in breathing, cough, compromised physical status, anxiety, and uncertainty. ^7, 9– 11^ Psychological distress is significantly correlated with COPD severity and poorer quality of life. Several studies have reported that patients with COPD have a higher prevalence of anxiety (21–96%) compared with the general population (20%). ^12– 14^ In addition, most of the patients diagnosed with moderate-to-severe COPD reported severe breathlessness, which is worsened with the experience of anxiety or depression. This may increase the frequency and severity of breathing symptoms, number of readmissions, and mortality. ^14– 16^ Further, several studies reported that anxiety was a predictor of low mean of total score of HRQoL. ^17– 21^ In Jordan, high prevalence of anxiety and depression, and poor quality of life were found among COPD patients and their partners. ^17^

Illness-related uncertainty is all about the patients’ inability to comprehend the process underpin their disease and being unable to expect the events associated with it. ^22^ Two studies have shown that greater uncertainty is associated with anxiety, impaired HRQoL, and fatigue, ^23, 24^ and another has shown that it impairs self-expectancy and resulted in maladaptation with the disease. ^25^ In a longitudinal study that was conducted to examine relationship between uncertainty and complexity of COPD patient outcomes, it was found that depression and anxiety were associated with an increased frequency of breathlessness and poor quality of life. ^26^ Limited studies have discussed uncertainty from COPD patients’ perspectives and its impact on HRQoL specifically. ^27^ Assessing uncertainty and anxiety and their effect on HRQoL among COPD patients would aid the effort of symptoms management among such patients. Thus, the aim of the study was to assess level of anxiety, uncertainty, and HRQoL among COPD patients in Jordan; and explore the relationships between these variables.

## Methods

A correlational design using a cross-sectional survey method was used. The sample size was estimated based on generic correlation test with a power of 80, 0.05 alpha, and a medium effect size (0.3). The minimum sample required was 82, however a sample of 153 COPD patients was included. Patients were included if they were 40 years and older and agreed to participate. Patients with primary diagnosis of bronchial asthma, bronchiectasis, and chronic obstructive asthma were excluded. The study was conducted at pulmonary clinics in three major referral hospitals in the capital, Amman. These clinics usually provide care for COPD patients from different parts of the country. Medical care and follow up is provided mainly by pulmonary medicine specialists in these clinics. Ethical approval to conduct the study was obtained from Institutional Review Board of the involved hospitals (IRB reference, 90/2019/724). Patients’ participation was voluntary and based on informed written consent.

### Data collection and instruments

Data were collected by one researcher for all patients using standardised structure interview in the period from July 2019 to January 2020. The data collection questionnaire included: sociodemographic and clinical characteristics part, HRQoL Questionnaire, State-Trait Anxiety Inventory, and Mishel’s Uncertainty of Illness Scale. Data were collected using a structured interview format by one trained researcher for all patients.

*Respiratory HRQoL Questionnaire-C version*

The Arabic version ^28^ of the St. George’s Respiratory Questionnaire, C version (SGRQ-C), was used to measure HRQoL among patients with respiratory disease like COPD and asthma. ^29^ The original SGRQ-C contains 17 items and three subscales that are: symptoms (items 1 to 8), activity-related (items 11 and 15), and impact-related (items 9, 10, 12 to 14, 16 and 17). Higher scores in the total SGRQ-C and sub-scales indicate a decrease in quality of life. The scale’s validity and reliability was established. ^30^ Cronbach’s alpha internal consistency of the scale was 0.95 and the long-term test–retest reliability ranged from 0.70 to 0.86. ^28, 30– 32^ The internal consistency (Cronbach’s alpha) of the Arabic version of SGRQ-C in this study was 0.83.

*Mishel’s uncertainty of illness scale*

Mishel’s uncertainty of illness scale (MUIS) was used to assess uncertainty related to disease trajectory. It consists of a 23-items using a five-point Likert scale (strongly agree to strongly disagree). The level of illness uncertainty increased as the total score increased. The uncertainty total score also can be divided into three levels: mild (23–53), moderate (54–84), and high (≥85). ^33^ The scale has a Cronbach alpha of 0.74–0.92. ^22, 24^ The Arabic version ^34^ was used in the study. Internal consistency (Cronbach’s alpha) of the Arabic version of MUIS-C in this study was 0.86.

*State-* Trait *Anxiety Inventory (SAI)*

The SAI ^35^ consists of 20 self-reported items that measure patients’ situational anxiety levels. The SAI has responses 1–4 where one means “not at all”, two “somewhat”, three “moderately so”, and four “very much so”. The total scores can range 20–80: a higher total score indicates increased level of anxiety. ^35^ The Cronbach’s alpha coefficient for the STAI was 0.92. ^35^ The internal consistency (Cronbach’s alpha) of the Arabic version of SAI in this study was 0.94. Permission to use the SAI was obtained from the copyright holder Mind Garden, Inc. ^36^

### Data analysis

The Statistical Package for Social Science ( **IBM SPSS Statistics, RRID:SCR_019096)** version 23 ^37^ for Windows was used for data analysis. Mean, frequency, standard deviation, minimum, and maximum were used to summarize study variables. The Pearson correlation coefficient was used to explore the relationship between HRQoL and other variables.

## Results

A total of 153 patients completed the study with no missing data. The mean (SD) age of participants was 66.8 (10.3) years. There were 144 (94.1%) male participants. 77 (50.3%) of participants were current smokers, and 68 (44.4%) were ex-smokers. Sociodemographic characteristics of participants are presented in Table 1.

**Table 1.** Sociodemographic characteristics of participants (N = 153).

Sociodemographic variables	N (%)	Mean (SD, Range)
Age 40–60 61–70 > 70	40 (26.1) 52 (34) 61 (39.9)	66.8 (10.3, 40–105)
Sex Male Female	144 (94.1) 9 (5.9)	
Marital status Married Not married	139 (90.8) 14 (9.2)	
Level of education Primary or below Preparatory up to secondary University and above	58 (37.9) 56 (36.6) 39 (25.5)	
Employment status Employed Not employed Retired	37 (24.2) 74 (48.2) 42 (27.5)	
Types of work Pollution-based exposure Non-pollution-based exposure Not worked	33 (21.6) 45 (29.4) 75 (49)	
Living status Alone With wife/husband only With one of the close family members Others	3 (2) 46 (30.1) 99 (64.7) 5 (3.3)	
Help and support provider Wife/husband Any of the family members Others None	89 (58.2) 56 (36.6) 2 (1.3) 6 (3.9)	
Smoking status Nonsmoker Current smoker Ex-smoker	8 (5.2) 77 (50.3) 68 (44.4)	
Age of beginning smoking *		16.8 (5.9, 2–54)
Smoke/years *		45.4 (11.8, 10–71)
Smoking pack. years **		51.5 (37.6, 1.9–178.5)
Type of hospitals Public University hospital	74 (48.4) 79 (51.6)	

M = Mean, SD = Standard Deviation, R = Range (Min – Max), % = Frequency.

* in the 146 current or past smokers.

** in the 77 current smokers.

The mean (SD) score of the SGRQ-C which represent the HRQoL was 57.9 (20.5) with a range of 6–92. The highest subscale scores were for the activity domain: 74.6 (21.9); and the lowest score was for the symptom’s domain: 43.8 (25.0). The results indicate that the level of HRQoL among patients with COPD in this study was reduced across all domains of HRQoL measurement.

The mean (SD) total score of the patients’ anxiety level was 38.1 (11.1), ranging 23–73, which indicates that the level of patients’ anxiety was moderate. The higher anxiety means (2.36, 2.36, 2.28, 2.25, and 2.18) were with items 15, 16, 10, 5, and 11, respectively, which are related to the positive response of patients feeling; and the lower anxiety means (1.33, 1.41, 1.41, 1.45, and 1.50) were with items 9, 17, 18, 13, and 3, respectively, which are related to the negative response of patients feeling. The mean responses to each item on the SAI scale are presented in Table 2.

**Table 2.** Description of Mean Anxiety Score (State Anxiety Scale) (N = 153).

Scale item	Mean (SD)
1. I feel calm	2.18 (0.8)
2. I feel secure	2.12 (0.8)
3. I am tense	1.50 (0.8)
4. I feel strained	1.51 (0.9)
5. I feel at ease	2.25 (0.8)
6. I feel upset	1.60 (0.9)
7. I am presently worrying over possible misfortunes	1.44 (0.8)
8. I feel satisfied	2.07 (0.7)
9. I feel frightened	1.33 (0.7)
10. I feel comfortable	2.28 (0.8)
11. I feel self-confident	2.18 (0.8)
12. I feel nervous	1.7 (1)
13. I am jittery	1.45 (0.7)
14. I feel indecisive	1.78 (0.9)
15. I am relaxed	2.36 (0.8)
16. I feel content	2.36 (0.8)
17. I am worried	1.41 (0.8)
18. I feel confused	1.41 (0.7)
19. I feel steady	1.97 (0.8)
20. I feel pleasant	1.84 (0.6)
Total mean	38.1 (SD, 11)

The mean (SD) total score of uncertainty was 66.1 (11.1) and ranged 31–94. According to the score categories proposed by, ^33^ the mean score of 66.1 in the current study indicates that the average patients’ uncertainty was moderate. The higher uncertainties (3.8, 3.7, and 3.7) were with items 13, 18, and 7, respectively, which are related to the ambiguity (cues about state of illness), and the lower uncertainties (1.8 and 2.1) were with items 8 and 6, respectively, which are related to the complexity (cues about treatment). Patient responses for the MUIS-C scale are presented in Table 3.

**Table 3.** Description of Mean Uncertainty Score (MUIS-C) (N = 153).

Scale item	Mean (SD)
1. I do not know what is wrong with me.	2.5 (1.1)
2. I have a lot of questions without answers.	2.9 (1.2)
3. I am unsure if my illness is getting better or worse.	3.1 (1.1)
4. It is unclear how bad my pain will be.	3.3 (1.1)
5. The explanations they give about my condition seem hazy to me.	2.76 (1.1)
6. The purpose of each treatment is clear to me.	2 (0.9)
7. My symptoms continue to change unpredictably.	3.67 (0.86)
8. I understand everything explained to me.	1.8 (0.7)
9. The doctors say things to me that could have many meanings.	2.79 (1)
10. My treatment is too complex to figure out.	2.6 (0.7)
11. It is difficult to know if the treatments or medications I am getting are helping.	2.7 (0.9)
12. Because of the unpredictability of my illness, I cannot plan for the future.	3.4 (1)
13. The course of my illness keeps changing. I have good and bad days.	3.8 (0.87)
14. I have been given many differing opinions about what is wrong with me.	2.72 (0.9)
15. It is not clear what is going to happen to me.	3.5 (0.9)
16. The results of my test are inconsistent.	3 (0.7)
17. The effectiveness of the treatment is undetermined.	2.9 (0.8)
18. Because of the treatment, what I can do and cannot do keeps changing.	3.67 (0.9)
19. I’m certain they will not find anything else wrong with me.	2.3 (1.2)
20. The treatment I am receiving has a known probability of success.	2.7 (0.7)
21. They have not given me a specific diagnosis.	2.47 (1)
22. The seriousness of my illness has been determined.	2.71 (1)
23. The doctors and nurses use everyday language so I can understand what they are saying.	2.45 (0.9)
Total mean	66.1 (SD, 11.1)

The Pearson correlation was utilized to test the relationship between the total HRQoL score and perceived anxiety score and the total uncertainty score. The results revealed the presence of a statistically significant positive relationship between HRQoL and anxiety ( *r* = 0.433, *p* < 0.01), and uncertainty ( *r* = 0.483, *p* < 0.01). The correlations between total HRQoL and subscales, with perceived anxiety and uncertainty are presented in Table 4.

**Table 4.** Correlations matrix between HRQoL, anxiety and uncertainty.

Variable	Symptom score total	Activity score total	Impact score total	HRQoL total score
	( *r*, *p*-value)	( *r*, *p*-value)	( *r*, *p*-value)	( *r*, *p*-value)
Perceived Anxiety	(0.427, <0.001)	(0.357, <0.001)	(0.388, <0.001)	(0.433, <0.001)
Uncertainty	(0.448, <0.001)	(0.344, <0.001)	(0.444, <0.001)	(0.483, <0.001)

## Discussion

In this study, more than one-third of the Jordanian patients reported a moderate level of anxiety. Our finding of anxiety level was in line with the findings of a systematic review which indicated that anxiety was prevalent among COPD patients and the anxiety level was moderate in several studies. ^38^ Anxiety is considered one of the comorbidities associated with physical and psychological discomfort. ^39^ Anxiety can become severe and prolonged and cause significant disruption for patients’ daily functioning and socialization. ^40^ It has been acknowledged that anxiety could increase COPD severity and interfere with patients’ daily life activities and then resulted in a poor quality of life. ^14– 16^ Anxiety doubles the likelihood of having limited daily living activity, exercise intolerance, and severe and frequent symptoms in COPD patients. The results of the current study are in line with the previous literature that found patients with a significant increase in the levels of anxiety are more likely to have poor HRQoL. ^18, 19, 21^ Anxiety disorders are disabling and, unless adequately treated, can become chronic, lower self-esteem, predispose to suicidal ideation, and increase the risk of hospitalization and ultimate worsen HRQoL. Healthcare providers should assess the level of anxiety on each patient’s visit and should promote interventions that can lower patient’s anxiety level.

The results of the study showed that Jordanian patients with COPD have experienced a moderate level of uncertainty concerning their disease and its management. This was consistent with the literature. ^23, 24, 34, 41, 42^ Uncertainty among COPD patients has been limitedly investigated. ^23, 43^ This study provides information about the experience of uncertainty among Jordanian COPD, and its relationships with anxiety and HRQoL. The current evidence showed that patients had relatively high levels of uncertainty and it affected their disease-related outcome. Indeed, Jordanian patients diagnosed with COPD reported a high level of uncertainty in most of the uncertainty items scale. For those patients, the uncertainty might generate some negative emotions including anxiety, unexpected feeling, frustrations, and nervousness behavior by delaying treatment plans and strategies; the treatment itself; the decision-making process; the patient's response to the therapy or complications; and ultimately the patients' overall HRQoL might also be compromised. The findings in our study are consistent with a study conducted by. ^26^ When disease-related symptoms are not adequately managed, uncertainty feeling increased combined with apprehension about disease perception and management. ^34, 44^ Jiang and He ^23^ reported that COPD used cognitive adaptation to the feeling of uncertainty. The cognitive–behavioral intervention is a useful example in strengthening health education and psychological support to decrease the uncertainty in illness, increase the quality of life, and for better coping among COPD patients with their disease problems. ^23, 43^ The adoption of such programs among Jordanian COPD patients would add more benefit in enhancing their quality of life and improve disease outcomes.

Jordanian patients with COPD reported poor HRQoL. This finding was consistent with the results from two previous studies. ^45, 46^ Also, the study was consistent with other studies conducted in different global countries, indicating that dyspnea, cough, and fatigue were the most burden symptoms affecting HRQoL. ^47– 50^ This result can be related to several factors: activity domains had the highest scores indicated the leading cause of poor quality of life is the factors related to the severity of the disease and level of activity. Also, the fact that most patients in this study were older people, their level of education from illiterate up to the primary, not employed, types of their work and having severe disease stages and being anxious and uncertain. Inconsistent with our findings, Sharma and Joshi ^51^ found that subscales' psychological and social domains had the highest scores.

The present study revealed the significant correlation between the perceived anxiety and HRQoL. The result was in the lines with the previous literature ^17, 18, 20, 21^ that revealed COPD patients with anxiety had a poor HRQoL and anxiety was the determinant of low HRQoL score. Studies conducted by Borge *et al.*; ^12^ Chen *et al.*; ^52^ and Lim *et al.* ^53^ reported a strong positive correlation between anxiety and HRQoL among COPD patients. The results of the present study indicated that a higher uncertainty score was correlated with poorer HRQoL. This might be because patients lack knowledge of the disease, noncompliance to treatment, and having vagueness about the disease's progress. Hoth *et al.* ^26^ suggested that social support might help in reducing uncertainty and improving quality of life. Therefore, it is necessary to reinforce social support for COPD patients to improve their coping strategies and social adaptability to improve their quality of life.

## Conclusions

Patients with COPD participated in the study in general have low quality of life, significant level of anxiety and uncertainty. The findings suggest that anxiety and uncertainty are correlated with poorer HRQoL among COPD patients. Healthcare providers need to pay more attention to assess anxiety and uncertainty components on a regular basis among COPD patients and institute appropriate management.

## Data Availability

Harvard Dataverse: Underlying data for ‘Health-related quality of life, uncertainty, and anxiety among patients with chronic obstructive pulmonary disease’, https://doi.org/10.7910/DVN/VWRTAW. ^54^ This project contains the following underlying data: socio-demographic and clinical variable, Uncertainty scale, anxiety scale, and St. George’s Respiratory Questionnaire. Data are available under the terms of the Creative Commons Zero “No rights reserved” data waiver (CC0 1.0 Public domain dedication).
