# Supplementary material for: Health-Related Quality Of Life, Uncertainty, ‎and Anxiety among Patients with Chronic Obstructive Pulmonary Disease
Source: F1000Res. 2021 May 26;10:420. [Version 1] doi: 10.12688/f1000research.51936.1 (PMC8577057; doi:10.12688/f1000research.51936.1)
Supplement: Peer review history

# Peer review: Reviewer response for version 1

**DOI:** 10.5256/f1000research.55148.r96908

**Published:** 2021-11-08

**Authors:** Abeer A. Selim

**Affiliations:** 1 College of Nursing - Riyadh, King Saud Bin Absulaziz University for Health Sciences, Riyadh, Saudi Arabia; 2 Faculty of Nursing, Psychiatric and Mental Health Department, Mansoura University, Mansoura, Egypt

The paper is addressing a significant research area which is quality of life and COPD along with anxiety and uncertainty.

- The paper needs English language editing.
- In the introduction, first paragraph, the authors focused on male Jordanian smokers, how about females?
- The design is cross-sectional, not correlational cross-sectional.
- Specify the type of COPD diseases included in the study.
- Provide rationales for including only 40 years old and more in the study.
- Data collection section: this statement is redundant "Data were collected using a structured interview format by one trained researcher for all patients".
- At the beginning of the discussion section, summarize the results, then interpret them.
- In the discussion, move the following statement to the conclusion section: "Healthcare providers should assess the level of anxiety on each patient’s visit and should promote interventions that can lower patient’s anxiety level."
- "The findings in our study are consistent with a study conducted by. ^26^" - this statement is not complete.
- Elaborate on patient's education about the disease, treatment, and prevention.
- Add limitations of the study.
- The paper is missing implications for practice.

Is the work clearly and accurately presented and does it cite the current literature?

Yes

If applicable, is the statistical analysis and its interpretation appropriate?

Yes

Are all the source data underlying the results available to ensure full reproducibility?

Yes

Is the study design appropriate and is the work technically sound?

Yes

Are the conclusions drawn adequately supported by the results?

Yes

Are sufficient details of methods and analysis provided to allow replication by others?

Yes

Reviewer Expertise:

Psychiatric and mental health nursing

I confirm that I have read this submission and believe that I have an appropriate level of expertise to confirm that it is of an acceptable scientific standard.

# Peer review: Reviewer response for version 1

**DOI:** 10.5256/f1000research.55148.r87506

**Published:** 2021-06-29

**Authors:** Nabeel Al-Yateem

**Affiliations:** 1 Department of Nursing, College of Health Sciences, University of Sharjah, Sharjah, United Arab Emirates

In general, the study description is very good. It is easy to read and follow. The study topic is important, and it has even more importance given the context of where it was conducted. In Jordan, the prevalence of COPD is high, as the authors truly reported. Therefore, more focus on the context of the study (i.e. in Jordan) would have been very beneficial as it would reinforce the importance of the study, and inform the reader about the current situation about COPD management and how this study would contribute to its improvement. This is also important given that in the Introduction, the authors cite a study conducted in Jordan that has addressed a similar topic (i.e. anxiety and depression, and quality of life among COPD patients and their partners).

 The Methods used in the study are quantitative, cross-sectional, and correlational design. It utilized known valid and reliable questionnaires. Given the stated aim of the study to “assess level of anxiety, uncertainty, and HRQoL among COPD patients in Jordan; and explore the relationships between these variables”, the methods and tools used seem appropriate. However, two main concerns related to the methods are:

- The small sample size, which is 153 participants. This might be relatively low for a correlational cross-sectional study, although the authors reported using a statistical calculator to determine the sample size.
- Another concern in the methodology is the use of 3 long questionnaires for the participants to complete. Also, all questionnaires are subjective in nature, it totally depends on participants' recall, perspectives, and subjective opinions.

The Results of the study are clear and well presented. The statistical analysis seems to be appropriate given the study aim, variables, and nature of the data collected.

 The Discussion is also fine, it compared the results of the current study with similar international literature. It was more descriptive and less critical in some places and may have lacked a focus on the implication of the results of the study to improve current practices. Given the experience of the researchers in the healthcare system in Jordan, I wish to see more discussion on how the results of this study can be translated into care elements to improve the service provided for COPD patients.

Is the work clearly and accurately presented and does it cite the current literature?

Yes

If applicable, is the statistical analysis and its interpretation appropriate?

Yes

Are all the source data underlying the results available to ensure full reproducibility?

Yes

Is the study design appropriate and is the work technically sound?

Yes

Are the conclusions drawn adequately supported by the results?

Yes

Are sufficient details of methods and analysis provided to allow replication by others?

Yes

Reviewer Expertise:

Nursing

I confirm that I have read this submission and believe that I have an appropriate level of expertise to confirm that it is of an acceptable scientific standard.

# Response

**Published:** 2021-06-30

**Authors:** Jafar Alshraideh

**Affiliations:** University of Jordan, Jordan

We wish to thank the reviewer for the valuable comments.
